# The associations of BMI, chronic conditions and lifestyle factors with insomnia symptoms among older adults in India

**DOI:** 10.1371/journal.pone.0274684

**Published:** 2022-09-15

**Authors:** T. Muhammad, Shivani Gharge, Trupti Meher

**Affiliations:** 1 Department of Family & Generations, International Institute for Population Sciences, Mumbai, Maharashtra, India; 2 Department of Bio-Statistics & Epidemiology, International Institute for Population Sciences, Mumbai, Maharashtra, India; University of Malaya, MALAYSIA

## Abstract

**Background:**

The aim of the study was to estimate the prevalence of insomnia symptoms and to examine the associations of body mass index (BMI), chronic diseases, and lifestyle factors with self-reported insomnia symptoms among older people in India.

**Methods:**

We conducted a cross-sectional study using data from the baseline wave of the Longitudinal Ageing Study in India (LASI) that was collected during 2017–18. A sample of 31,358 older adults aged 60 and above was included in the analyses. Descriptive statistics and bivariate and multivariable analyses were performed to obtain the results.

**Results:**

In this study, insomnia symptoms were reported by around 36 percent of older adults aged 60 and above. After controlling for socio-demographic factors, insomnia symptoms were positively associated with the risk of being underweight [AOR: 1.289, CI: 1.211–1.372] and negatively associated with obesity/overweight [AOR: 0.928, CI: 0.872–0.990] as compared to older adults with normal BMI. The odds of insomnia symptoms were higher among those who reported the following chronic conditions, i.e., hypertension [AOR:1.356, CI:1.278–1.438], diabetes [AOR:1.160, CI:1.074–1.254], chronic lung diseases [AOR:1.485, CI:1.351–1.632], bone-related diseases [AOR:1.561, CI:1.458–1.670] and any psychiatric disorders [AOR:1.761, CI:1.495–2.074]. In addition, older adults who were physically active [AOR: 0.850, CI:0.804–0.900] were less likely to report insomnia symptoms.

**Conclusions:**

The study suggests a high prevalence of insomnia symptoms among the older population in India. Early identification of the signs of insomnia in older population is crucial, as is timely treatment for any kind of sleep problems. In addition, nutrition-based interventions and individual disease-specific management programs may help minimize the stressful situations in later life and develop a good night’s sleep for the older population.

## Background

Insomnia has a significant impact on older people all over the world [1–6]. A general definition of insomnia is the lack of satisfaction with either the quality or quantity of sleep. This is frequently related to any or all of the following types of sleep problems: (1) difficulty falling asleep, (2) difficulty maintaining sleep, which is defined by frequent awakenings or issues falling back asleep following awakenings, and (3) early morning awakening with difficulty falling back asleep [7]. Insomnia can lead to increased risk of falls, sleepiness, daytime fatigue, difficulty in concentration and memory, and psychiatric disorders, thereby affecting an individual’s health, quality of life, everyday functioning, and economic conditions [8–11].

Old age alone does not predict incident reports of insomnia [1, 12]. Rather, because of the related comorbidities or chronic illnesses that are widespread in old age, the prevalence of insomnia and other sleep disorders is high in the older population [13–15]. Depression, anxiety, heart illness, diabetes, higher body mass index (BMI), and illnesses that cause discomfort and agony, such as arthritis, are all typical sleep disruptors among older people [16–20]. Multiple studies [15, 21–23] have found that lifestyle changes, as well as environmental and socioeconomic factors, are associated with sleep disturbances.

There has been extensive research conducted all over the world on the prevalence of insomnia and its correlates in elderly people [2, 13, 24–26]. In India, a household survey in urban West Bengal found that the prevalence of insomnia was around 15 percent among the older adult population [27], and around 13 percent of the 92 elderly participants in a cross-sectional study in Bangalore were found to have insomnia [28]. In South India, among those who reported having insomnia symptoms, "18 percent had trouble falling asleep, 18 percent had trouble staying asleep, and 7.9 percent suffered early morning awakenings," according to the survey results [29]. Other studies related to insomnia performed in Uttar Pradesh, Karnataka and Kerala employed a small sample size and had a narrow emphasis on hospital visitors [30–32]. Moreover, research has found links between multiple chronic conditions, sleep length, and sleep quality in older individuals. Yet, very few studies have examined these links in low-income nations [18], and even fewer have evaluated the relationship between obesity risks and insomnia symptoms. Thus, at a national level, studies on the prevalence of insomnia symptoms and their association with BMI, chronic conditions, and lifestyle factors in the Indian older population are very limited.

The aim of this study was to examine the associations of obesity, chronic conditions, and lifestyle factors with insomnia symptoms among a community-dwelling older population by utilizing data from the longitudinal aging study in India (LASI). The study hypothesized that a higher BMI, chronic conditions, and adverse lifestyle habits, which are potentially modifiable risk factors, are positively associated with insomnia symptoms in later years of life. The study employed large-scale survey data, with several socio-demographic, lifestyle, and medical characteristics focusing on a specific age group, in a resource-constrained setting with limited previous literature.

## Methods

### Data

We used data drawn from the baseline wave of the LASI survey (2017–18) which was a nationally representative survey of scientific investigation of the health, economics, and social determinants and consequences of population aging in India. The survey was conducted under the stewardship of the Ministry of Health and Family Welfare (MoHFW), Government of India, and coordinated by the International Institute for Population Sciences (IIPS), National Programme for Health care of Elderly (NPHCE), Harvard T. H. Chan School of Public Health (HSPH) and the University of Southern California (USC). LASI is the first longitudinal aging study in India that provides national and state-level data on aging, chronic health conditions, functional health, mental health, symptom-based health conditions, health insurance and healthcare utilization, biomarkers, welfare programs, work and employment, retirement, family and social networks, satisfaction, and life expectations. The LASI was designed to simultaneously generate data, raise awareness about the health issues of older people, and inform public policies in India and its states [33].

LASI adopted a multistage stratified area probability cluster sampling design with the unit of observation being older adults aged 45 and above and their spouses irrespective of age. The survey utilizes a three-stage sampling design for rural areas and a four-stage sampling design for urban areas within each state. In the first stage, Primary Sampling Units (PSUs) were selected, which were the sub-districts, and in the second stage, villages in rural areas and wards in urban areas were selected from the selected PSUs. In the third stage, 32 households were selected from each selected village in rural areas, whereas in the case of urban areas, one Census Enumeration Block (CEB) was randomly selected from each urban area, following which 35 households were selected from each selected CEB. This survey collected information from a nationally representative sample of 65,506 households with 72,250 individuals aged 45 and above and their spouses, including 31,464 older adults aged 60 years and above and 6,749 oldest-old individuals aged 75 years and above across 35 states and union territories of India [33].

### Variable description

#### Outcome variable

The respondents were screened for insomnia symptoms through a series of questions in the LASI questionnaire. Individuals were asked (i) how often they had trouble falling asleep, (ii) how often they woke up during the night and had trouble getting back to sleep, and (iii) how often they woke up too early in the morning and were unable to fall asleep again. These three questions were chosen to assess insomnia symptoms based on previous studies that have used similar criteria [11, 34, 35]. Accordingly, difficulty in initiating sleep (DIS), difficulty in maintaining sleep (DMS), and early morning awakening (EMA) were analyzed. Responses included ‘never’, ‘rarely’ (1–2 nights per week), ‘occasionally’ (3–4 nights per week), and ‘frequently’ (5 or more nights per week). For the current study, we defined individuals as experiencing insomnia symptoms if they answered ‘occasionally’ or ‘frequently’ to at least one of the three mentioned questions.

### Explanatory variables

#### Health factors

- *Body mass index (BMI)*: BMI was calculated by dividing body weight by body height squared, and the results were classified as ‘underweight’ (< 18.5 kg/m^2^), ‘normal’ (18.5–24.9 kg/m^2^) and ‘overweight/obese’ (≥25 kg/m^2^) [36].
- *Hypertension*: Individuals were asked whether they had been diagnosed with hypertension or high blood pressure by any health professional, and the responses were coded as ‘no’ or ‘yes’.
- *Diabetes*: Individuals were asked whether they had been diagnosed with diabetes or high blood sugar by any health professional, and the responses were coded as ‘no’ or ‘yes’.
- *Lung disease*: This information was derived from a question on whether a person was ever diagnosed with a chronic lung disease such as asthma, chronic obstructive pulmonary disease, chronic bronchitis, or other chronic lung problems by any health professional. The responses were coded as ‘no’ or ‘yes’.
- *Bone-related diseases*: Individuals were asked whether they had been diagnosed with arthritis or rheumatism, osteoporosis or other bone/joint diseases by any health professional, and the responses were coded as ‘no’ or ‘yes’.
- *Psychiatric disorder*: This information was derived from a question on whether a person was ever diagnosed with any neurological or psychiatric problems such as depression, Alzheimer’s/Dementia, unipolar or bipolar disorders, convulsions, Parkinson’s etc. by any health professional. The responses were coded as ‘no’ or ‘yes’.

### Lifestyle factors

- *Food sufficiency*: This information was obtained from three questions that were asked of the respondents on household food availability with a reference period of 12 months. The questions were on (i) ever reduce the size of the meal or skip meal; (ii) hungry but didn’t eat; (iii) not eat for a whole day because of insufficiency of food at the household. The responses were coded as ‘yes’ or ‘no’.
- *Using tobacco*: This provides information on whether a person is still using smoke/smokeless tobacco product or not. Those who were still using it were coded as ‘yes’ and those who never used it or have quit were coded as ‘no/quit’.
- *Drink alcohol*: This information was obtained from the question “In the past three months, on an average, how frequently (on how many days), have you had at least one alcoholic drink?” Those who answered as “none” were coded as ‘no’ and other responses were recoded as ‘yes’.
- *Social participation*: Social participation was measured through the question “Are you a member of any social organizations, religious groups, clubs, or societies?” And it was coded as ‘no’ and ‘yes’.
- *Physical activities*: This information was assessed from the question “How often do you take part in sports or activities that are moderately energetic such as, cleaning house, washing clothes by hand, fetching water or wood, drawing water from a well, gardening, bicycling at a regular pace, walking at a moderate pace, dancing, floor or stretching exercises?” Physical activity status was recoded as ‘yes’ for responses like ‘every day’ and ‘more than once a week.’ However, responses like, ‘once a week’, ‘one to three times in a month’ and ‘never’ were recorded as ‘no’.

### Socio-demographic factors

- *Age*: Individuals were asked about their current age at the time of the survey. In this study, age was classified into 3 groups as ‘60–69 years’, ‘70–79 years’ and ‘80+’.
- *Sex*: It was coded as male and female.
- *Marital status*: It was recoded as ‘currently married’, ‘widowed’ and ‘others’ (divorced/separated/deserted/never married).
- *Education*: This variable describes the educational level of the respondents. It was recoded as ‘no education’, ‘primary’, ‘secondary’ and ‘higher’.
- *Working status*: The respondents were asked about whether they are currently working or not during the time of the survey. Based on their responses, this variable was coded as ‘no’ and ‘yes’
- *Caste*: It was recoded as ‘Scheduled Caste/Scheduled Tribe’ (SC/ST), ‘Other Backward Class’ (OBC), and ‘Others’ [37].
- *Religion*: This variable was recoded as ‘Hindu’, ‘Muslim’, ‘Christian’ and ‘Others’.
- *Monthly per capita expenditure (MPCE) quintile*: It was assessed using household consumption data. A set of 11 questions on the expenditures on food items and 29 questions on expenditures on non-food items were used to canvass the sampled households. Here the variable was coded as ‘poorest’, ‘poorer’, ‘middle’, ‘richer’ and ‘richest’.
- *Place of residence*: It was coded as ‘rural’ and ‘urban’.
- *Living arrangements*: Individuals were asked about whether they were living alone or with their children, spouse or others, and based on their responses, this variable was recoded into two categories as ‘living alone’ and ‘living with spouse/children/others’.
- *Regions*: The regions of India were coded as ‘North’, ‘Central’, ‘East’, ‘Northeast’, ‘West’, and ‘South’ [36].

### Statistical analyses

All statistical analyses were carried out using Stata version 14. Bivariate analyses were performed to estimate the prevalence of insomnia symptoms among individuals aged 60 and over in various states/UTs and regions of India as well as for the categories of covariates. Chi-square tests were used to determine whether independent variables had significant associations with the outcome variable at p < .05. Three separate multivariate models were then fitted to discern the extent to which health factors alone, and in combination with lifestyle and demographic/socioeconomic factors, explain the associations with insomnia symptoms.

The binary logistic regression model is usually put into a more compact form as follows:

$$Logit[P(Y=1)]=\beta_{0}+\beta *X$$

The parameter *β*_0_ estimates the log odds of the insomnia symptoms for the reference group, while *β* estimates the maximum likelihood, the differential log odds of the insomnia symptoms associated with the set of predictors X, as compared to the reference group.

Model 1 is controlled for all the health-related variables, whereas, Model 2 is controlled for variables in Model 1 and lifestyle factors. Finally, Model 3 is controlled for variables in Model 2 and all demographic and socioeconomic factors.

## Results

Table 1 presents the prevalence of insomnia symptoms defined in terms of difficulty initiating sleep (DIS), difficulty maintaining sleep (DMS), and early morning awakening (EMA) among older adults aged 60 and above. Around 36.3 percent of older adults reported at least one of the three insomnia symptoms. However, 21.2 percent suffered from DIS, 24.9 percent experienced DMS and 24 percent went through EMA. The prevalence of insomnia symptoms ranged from 32 percent to 37 percent across all the regions in India, the highest cases being recorded in the Central (37.3 percent) and Northern (37 percent) regions. The majority of the older adults experiencing insomnia symptoms were from the states/ UTs of Himachal Pradesh (52.1 percent), Uttarakhand (49.1 percent), and Delhi (47.1 percent). The lowest prevalence of insomnia symptoms was recorded in Lakshadweep (12.1 percent), followed by Nagaland (12.4 percent) and Manipur (18.6 percent).

**Table 1:** Prevalence of insomnia symptoms among individuals aged 60 years and above by states and UTs.

States	Insomnia symptoms (%)	DIS (%)	DMS (%)	EMA (%)	N
**North**	**37.0 **	**20.7 **	**25.9 **	**23.4 **	**5,812 **
Jammu & Kashmir	33.5	18.3	21.2	19.7	731
Himachal Pradesh	52.1	32.2	36.7	31.5	621
Punjab	36.2	19	24.9	23.7	1,004
Chandigarh	32.5	16.7	20.6	19.6	394
Uttarakhand	49.1	26.4	35.6	31.8	641
Haryana	46.2	25.3	28.9	30.7	848
Delhi	47.1	21.9	35	29.9	495
Rajasthan	28.5	17.5	21.5	17.5	1,078
**Central**	** 37.3**	**20.4 **	**25.2 **	**27.0 **	**4,262 **
Chhattisgarh	38.0	15.9	22.8	24.9	780
Madhya Pradesh	40.6	22.5	29.6	29.5	1,313
Uttar Pradesh	35.4	19.8	23.0	25.9	2,169
**East**	**36.4**	**23.8**	**23.9**	**24.2**	**5,757**
Bihar	37.0	23.4	22.3	24.2	1,808
Jharkhand	28.8	17.6	19.2	20.4	1,168
Odisha	30.4	16.8	20.9	20.6	1,237
West Bengal	41.0	29.6	28.4	27.1	1,544
**Northeast**	**32.0 **	**17.1 **	**22.5 **	**20.3 **	**3,752**
Assam	36.8	18.3	27.1	25.7	816
Arunachal Pradesh	36.2	17.5	26.6	19.7	318
Manipur	18.6	13.2	11.4	10.1	606
Meghalaya	24.7	9.7	15.3	9.4	412
Mizoram	31.7	16.5	17.7	13	531
Nagaland	12.4	8.8	4.7	5.3	608
Tripura	23.5	19.8	14.6	9.6	461
**West**	**36.2**	**20.3**	**24.1**	**22.1**	**4,303 **
Daman & Diu	38.3	24.0	28.1	23.8	434
Dadra & Nagar Haveli	29.9	13.4	16.2	18.5	451
Gujarat	33.4	18.8	24.6	19.5	991
Goa	30.7	21.7	19.8	15.7	637
Maharashtra	37.4	20.9	23.9	23.2	1,790
**South**	**35.6**	**20.6**	**26.2**	**23.4**	**7,578 **
Andaman & Nicobar Islands	38.9	24.6	28.5	26.5	523
Andhra Pradesh	31.1	18.1	22.2	21.9	1,105
Kerala	35.8	26.9	25.4	18.7	1,209
Karnataka	41.9	21.9	35.1	29.2	1,004
Lakshadweep	12.1	9.8	7.9	6.8	502
Puducherry	42.1	26.7	27.2	26.6	640
Tamil Nadu	32.6	18.9	21.1	19.5	1,534
Telangana	34.1	20.5	23.5	24.6	1,061
**India**	**36.3**	**21.2**	**24.9**	**24.0**	**31,464**

**Note:** DIS- Difficulty initiating sleep; DMS- Difficulty maintaining sleep; EMA- Early morning awakening

Table 2 demonstrates the bivariate distribution of insomnia symptoms among older adults aged 60 and above by selected background characteristics. Insomnia symptoms differed by age, gender, working status, education, marital status, living arrangement, all health conditions, food sufficiency, alcohol consumption, social participation, and physical activity. The percent of insomnia symptoms among older adults was higher in the age group of 80 and above (40.9 percent), females (40.3 percent), widowed (40.1 percent), non-educated (39.6 percent), non-working older population (39.3 percent), rural residence (37.3 percent), and among those who were living alone (45 percent). Further, considering the health factors, a higher prevalence estimate of insomnia symptoms was also observed among older adults who were underweight (40 percent), suffering from hypertension (41.6 percent), chronic lung diseases (46.7 percent), bone-related diseases (47.4 percent), and those experiencing psychiatric disorders (51.9 percent). Insomnia symptoms were reported high among older adults who had insufficient food (52.3 percent), not consuming tobacco (37 percent) and alcohol (36.8 percent), socially active (37.1 percent), and not indulging into physical activities (39.4 percent).

**Table 2:** Percentage of individuals aged 60 years and above with insomnia symptoms by socio-demographic, health and life-style factors.

Variables	Insomnia symptoms	DIS	DMS	EMA	N
**Socio-demographic factors**					
**Age group** *					
60–69	34.4	19.4	23.3	22.0	18,974
70–79	38.4	22.8	26.5	25.9	9,101
80+	40.9	26.1	29.2	29.8	3,389
**Sex** *					
Male	31.9	17.8	21.7	21.1	15,098
Female	40.3	24.4	27.8	26.6	16,366
**Marital status** *					
Currently married	34.3	19.4	23.1	22.2	19,920
Widowed	40.1	24.2	28.2	27.6	10,719
Others	32.3	19.7	20.3	16.7	825
**Education** *					
No education	39.6	23.3	27.4	27.1	16,888
Primary	35.2	19.7	24.2	22.4	7,560
Secondary	28.7	17.1	18.7	17.1	4,614
Higher	27.7	16.3	19.2	17.4	2,401
**Working status** *					
No	39.3	23.4	27.5	26.5	22,142
Yes	29.7	16.1	12.1	18.6	9,307
**Caste** *					
SC/ST	36.8	21.8	25.2	24.8	10,313
OBC	36.7	20.9	25.3	24.3	11,886
Others	35.2	20.6	23.8	22.6	8,218
**Religion** *					
Hindu	36.6	21.0	25.2	24.1	23,037
Muslim	36.4	23.4	24.5	25.4	3,731
Christian	28.0	17.4	18.9	17.3	3,150
Others	36.6	21.5	24.0	23.9	1,545
**MPCE quintile** *					
Poorest	36.9	21.0	25.1	25.9	6,484
Poorer	35.8	22.1	24.3	23.7	6,477
Middle	36.2	21.3	24.7	23.4	6,416
Richer	35.8	20.2	24.4	23.2	6,170
Richest	37.2	21.2	26.3	23.7	5,917
**Place of residence** *					
Rural	37.3	21.5	25.2	24.9	20,725
Urban	34.1	20.5	24.3	21.9	10,739
**Living arrangement** *					
Living alone	44.6	26.7	33.0	29.5	1,622
With spouse/children/others	35.8	20.8	24.4	23.7	29,842
**Health factors**					
**Body mass index (BMI)** *					
Normal	35.1	19.6	23.6	22.6	14,708
Underweight	40.0	23.3	28.0	27.0	6,524
Overweight/Obese	34.5	21.1	23.6	22.1	6,818
**Hypertension** *					
No	33.8	19.1	22.9	22.3	20,387
Yes	41.6	25.3	29.0	27.6	10,995
**Diabetes** *					
No	36.4	21.3	25.0	24.2	26,521
Yes	35.9	20.6	24.5	23.2	4,860
**Chronic lung diseases** *					
No	35.4	20.3	24.1	23.2	29,013
Yes	46.7	30.3	33.6	32.7	2,370
**Bone related diseases** *					
No	33.6	19.1	22.7	22.1	25,799
Yes	47.4	29.7	34.1	31.7	5,585
**Psychiatric disorders** *					
No	35.9	20.7	24.5	23.6	30,538
Yes	51.9	36.7	39.2	37.3	843
**Lifestyle factors**					
**Food sufficiency** *					
Yes	34.8	20.1	23.9	22.8	28,932
No	52.3	32.4	35.7	36.5	2,257
**Using tobacco** *					
No/quit	37.0	21.9	25.5	24.3	21,357
Yes	34.8	19.7	23.7	23.4	9,841
**Drink alcohol** *					
No/quit	36.8	21.6	25.3	24.3	28,577
Yes	31.2	15.5	20.6	20.7	2,883
**Social participation** *					
No	36.3	21.2	25.0	24.1	28,885
Yes	37.1	21.0	22.9	21.0	2,128
**Physical activities** *					
No	39.4	23.3	27.7	27.1	14,586
Yes	33.7	19.3	22.5	21.4	16,878

Note:

* refers to < 0.05 level of significance; DIS- Difficulty initiating sleep; DMS- Difficulty maintaining sleep; EMA- Early morning awakening

For some variable the ‘N’ is not additive to the total ‘N’ because of and missing cases.

Table 3 provides estimates from the regression models evaluating the adjusted associations between insomnia symptoms and selected background characteristics distinguished into three models. Model 1 controlled for health characteristics and revealed that BMI, hypertension, chronic lung diseases, bone-related diseases and psychiatric problems were significantly associated with insomnia symptoms among older adults aged 60 and above. The likelihood of older adults experiencing insomnia symptoms was higher among those who were underweight [AOR: 1.289, CI: 1.211–1.372] and lower among those who were obese/overweight [AOR: 0.928, CI: 0.872–0.990] as compared to older adults with normal BMI. Older adults suffering from hypertension [AOR: 1.422, CI: 1.345–1.502], chronic lung diseases [AOR: 1.527, CI: 1.394–1.672], bone related diseases [AOR: 1.654, CI: 1.552–1.763] and psychiatric disorders [AOR: 1.787, CI: 1.528–2.088] were more likely to report insomnia symptoms.

**Table 3:** Adjusted odds ratio (AOR) for the association of covariates with reporting insomnia symptoms.

Background characteristics	AOR (95% CI)		
	Model 1	Model 2	Model 3
**Body mass index (BMI)**			
Normal	Ref.	Ref.	Ref.
Underweight	1.289*** (1.211–1.372)	1.259*** (1.181–1.341)	1.145*** (1.072–1.224)
Overweight/Obese	0.928* (0.872–0.990)	0.938 (0.879–1.001)	0.955 (0.892–1.022)
**Hypertension**			
No	Ref.	Ref.	Ref.
Yes	1.422*** (1.345–1.502)	1.412*** (1.334–1.492)	1.356*** (1.279–1.438)
**Diabetes**			
No	Ref.	Ref.	Ref.
Yes	1.036 (0.964–1.114)	1.040 (0.967–1.119)	1.159*** (1.072–1.252)
**Chronic lung diseases**			
No	Ref.	Ref.	Ref.
Yes	1.527*** (1.394–1.672)	1.519*** (1.386–1.665)	1.482*** (1.348–1.629)
**Bone related diseases**			
No	Ref.	Ref.	Ref.
Yes	1.654*** (1.552–1.763)	1.627*** (1.526–1.736)	1.556*** (1.454–1.665)
**Psychiatric disorders**			
No	Ref.	Ref.	Ref.
Yes	1.787*** (1.528–2.088)	1.747*** (1.492–2.045)	1.750*** (1.486–2.061)
**Food sufficiency**			
Yes		Ref.	Ref.
No		2.111*** (1.922–2.317)	2.096*** (1.902–2.309)
**Using tobacco**			
No/quit		Ref.	Ref.
Yes		0.980 (0.926–1.037)	1.068* (1.004–1.137)
**Drink alcohol**			
No/quit		Ref.	Ref.
Yes		0.903* (0.825–0.988)	1.069 (0.972–1.176)
**Social participation**			
No		Ref.	Ref.
Yes		0.871** (0.788–0.963)	1.114* (1.000–1.240)
**Physical activities**			
No		Ref.	Ref.
Yes		0.830*** (0.789–0.873)	0.813*** (0.770–0.859)
**Age group**			
60–69			Ref.
70–79			1.036 (0.975–1.102)
80+			1.079 (0.982–1.86)
**Sex**			
Male			Ref.
Female			1.338*** (1.250–1.432)
**Marital status**			
Currently married			Ref.
Widowed			1.136*** (1.066–1.211)
Others			0.999 (0.843–1.185)
**Education**			
No education			Ref.
Primary			0.965 (0.901–1.033)
Secondary			0.852*** (0.779–0.931)
Higher			0.700*** (0.618–0.793)
**Working status**			
No			Ref.
Yes			0.842*** (0.789–0.898)
**Caste**			
SC/ST			Ref.
OBC			1.161*** (1.086–1.241)
Others			1.086* (1.007–1.173)
**Religion**			
Hindu			Ref.
Muslim			0.808*** (0.740–0.882)
Christian			0.751*** (0.669–0.843)
Others			0.823** (0.724–0.936)
**MPCE quintile**			
Poorest			Ref.
Poorer			1.041 (0.935–1.100)
Middle			1.001 (0.921–1.087)
Richer			1.030 (0.946–1.120)
Richest			1.090 (0.997–1.191)
**Place of residence**			
Rural			Ref.
Urban			0.844*** (0.792–0.899)
**Living arrangement**			
Living alone			Ref.
With spouse/children/others			0.892 (0.790–1.007)
**Region**			
North			Ref.
Central			0.911 (0.829–1.002)
East			0.751*** (0.688–0.821)
Northeast			0.628*** (0.557–0.708)
South			0.687*** (0.630–0.750)
West			0.791*** (0.719–0.870)

**Note:** Ref.: reference category

*, **, *** refers to < 0.05, < 0.01 and < 0.001 level of significance

Model 2 controlled for health characteristics along with lifestyle factors and depicted health conditions along with food and lifestyle characteristics. Compared to older adults having sufficient food or food security, the odds of suffering from insomnia symptoms were higher among older people who reported food insufficiency [AOR: 2.111, CI: 1.922–2.3217]. Older adults consuming alcohol [AOR: 0.903, CI: 0.825–0.988] were less likely to experience insomnia symptoms as compared to those who did not consume alcohol. Older adults who were socially [AOR: 0.871, CI: 0.788–0.963] and physically [AOR: 0.830, CI: 0.789–0.873] active were less prone to insomnia symptoms as compared to those who were socially and physically inactive.

Model 3 after controlling for variables in Model 2 and all the socio-demographic characteristics revealed that most of the associations remained same. The association between insomnia symptoms and BMI, hypertension, lung diseases, bone-related diseases and physical activity decreased in magnitude but remained statistically significant in Models 2 and 3. The association between insomnia symptoms and psychiatric disorders decreased in magnitude in Model 2 and increased in magnitude in Model 3. However, it remained significant. The association between insomnia symptoms and diabetes was significant in Model 3, whereas insignificant in Models 1 and 2. Older females [AOR: 1.338, CI: 1.250–1.432] and widowed [AOR: 1.136, CI: 1.066–1.211] older individuals had higher odds of experiencing insomnia symptoms compared to their counterparts. Those having secondary [AOR: 0.852, CI: 0.779–0.931] and higher [AOR: 0.700, CI: 0.618–0.793] education were also less likely to have insomnia symptoms as compared to those who were not educated. Currently working [AOR: 0.842, CI: 0.789–0.898] older adults had lower odds of reporting insomnia symptoms as compared to non-working older adults. Urban [AOR: 0.844, CI: 0.792–0.899] older adults were less likely to have insomnia symptoms as compared to the rural older population.

## Discussion

This analysis of a large Indian older population found that 36.3 percent of them had experienced at least one type of insomnia symptom in the month prior to being interviewed. The prevalence of insomnia symptoms among older adults is higher than in previous Indian studies (13 to 17.7 percent) [3, 8], but slightly lower than the estimated figure in a study of older adults aged 65 and up living in catchment areas in low and middle-income countries (37.7 percent in India) [26]. Previous studies in other countries, however, have reported a higher prevalence of insomnia symptoms. A study in Greece, for example, discovered that 64.6 percent of community-dwelling older people aged 60–100 years had one or more insomnia-like symptoms [38]. Another study among U.S. older adults aged 65 and above found a proportion of 54.8 percent reporting at least one insomnia symptom [39]. A large proportion of older Italian adults (84.7 percent) also reported insomnia symptoms in a population-based study [40]. The lower prevalence may be attributed to the differences in the definitions of insomnia symptoms as well as methodological differences in the data collected from the clinical and general population samples.

Importantly, in our study, a statistically significant sex difference was found, and older women (40.3 percent) reported more insomnia symptoms than older men (31.9 percent). This finding is confirmed by previous literature suggesting that the prevalence of insomnia was significantly higher among females than males [41]. The possible reasons for this differential are often suggested as social and biological differences in terms of estrogen deficiency, prior psychological disorders, and caregiving roles [42–45]. Another study concluded that women may have specific predisposition factors of multiple insomnia symptoms than men which may involve both behavioral (such as adherence to specific diets and/or alcohol intake) and hormonal factors (such as a higher BMI and the use of hormonal replacement therapy) [46].

Compared to younger ages, sleep tends to be shallow, fragmented, and variable in duration in the middle and older ages [22]. The oldest-old participants in the present study reported higher rates of all types of insomnia symptoms. The finding is in accordance with previous studies, which suggested a decreased total sleep time with increasing age [47, 48]. It also supports the notion that sleep efficiencies, which are defined as the amount of sleep relative to the amount of time in bed, decrease with age [49]. However, researchers have argued that insomnia symptoms are a result of the decreased ability to maintain sleep in later years of life rather than a result of increased age itself [1]. Among the more common reasons for this decreased ability are chronic diseases and the medications used to treat those ill-health conditions [12]. Population-based studies have also shown that poorer health conditions are associated with sleep disturbances regardless of socioeconomic characteristics, which is in line with earlier studies [19, 50].

Furthermore, older adults who reported insomnia symptoms in the present study were more likely to have a range of chronic physical and mental illnesses such as hypertension, diabetes, lung disease, bone-related problems, and psychiatric disorders. The coexistence of sleep-disordered breathing in conditions such as chronic lung disease and diabetes may explain the associations between insomnia symptoms and chronic conditions [9, 49]. Consistent with multiple previous studies, we found insomnia symptoms among older adults aged 60 years and above to be associated with being hypertensive and diabetic [48, 51–53]. Furthermore, lung disease and bone-related diseases were also related to insomnia symptoms in older participants of this study. Concordantly, chronic musculoskeletal pain has been shown as a significant factor associated with insomnia and longitudinally predicted the severity of insomnia in older individuals [54]. Thus, the current study suggests that such diseases disrupt sleep by multiple mechanisms, such as pain, respiratory disorders, and the effects of medical treatments [55]. Although medical disorders can cause insomnia symptoms, sleep disturbances, as in reverse causation, can contribute to the severity of many of the disorders that cause insomnia [53].

The significant association of psychiatric disorders with insomnia symptoms in the present study corroborates earlier research where depression has been seen to have a positive correlation with sleep disorders [30, 56]. Also, a longitudinal study among Korean older adults aged 65 years and above, found baseline insomnia to be independently associated with depression and an increased number of physical disorders [57]. In most cases, psychiatric disorders are expressed as somatic complaints and may be overlooked as a normal part of the aging process and often go unrecognized by non-psychiatric physicians [58]. Similarly, as demonstrated in multiple studies, insomnia symptoms may be associated with under-detection of depression among older people [59, 60]. Hence, there is a need for a more in-depth examination of this association to come up with better policy measures and thus to prevent poor mental health adversely affecting the sleep habits of older individuals.

The effects of nutrient intake and dietary patterns on insomnia symptoms are well-documented in earlier studies [61, 62]. Importantly, in agreement with this, the results showed that older individuals who were underweight and those who reported insufficient food availability had more insomnia symptoms compared to older participants with a normal BMI and those who had sufficient food, respectively. This is supported by the previous findings that underweight populations had significantly lower quality of life scores compared to normal weight individuals [63, 64]. These findings are particularly important in terms of special socioeconomic and cultural characteristics of India, and thus, nutritional interventions should target middle-aged and older adults with limited economic resources in availing healthy food and eliminating the issue of malnutrition, and ultimately addressing their physical and psychological ill-being [65–67].

However, the association between overweight/obese and insomnia symptoms was statistically not significant in our study, which is contrary to previous findings on the increased risk of chronic insomnia among older people with obesity [68, 69]. The lack of statistical significance in the relationship between overweight and insomnia symptoms in this study may be due to lower rates of obesity and low BMI of the sample of older Indian adults in comparison to previous literature. The finding was, however, consistent with a meta-analysis showing that the odds of developing future insomnia among those who had obesity were not significantly greater than those who were normal-weight [70]. On the other hand, the lower prevalence of insomnia symptoms among overweight/obese older adults in our study may be explained by the possible reverse causality. For example, a review of findings suggested that personality traits such as perfectionism and a higher concern about health status among insomnia patients may be related to a healthier lifestyle preventing obesity [71]. Awareness programs that promote behavior norms through sleep education are important long-term solutions for dealing with insomnia symptoms caused by unhealthy behavioral changes in older adults.

When health conditions were controlled for, physical activity, and social participation were significantly negatively associated with insomnia symptoms that are consistent with earlier findings [58, 72–74]. However, the adverse consequences of sleep disorders on a person’s capacity for physical activity and the resultant injuries due to prolonged exercise are also discussed in the previous literature [75]. Thus, further investigation is required for a better understanding of the effect of physical activity on the quantity and quality of sleep and its impact on the fundamental physiology of the older population. Furthermore, in the adjusted multivariable model, there was no significant association between alcohol drinking and insomnia symptoms in our study. This finding is contradictory to previous literature relating alcohol use to faster initiation of sleep but significant sleep fragmentation [76, 77].

Additional diseases and other factors not measured in this study may also possibly be playing a role in the prevalence of insomnia symptoms. Due to the cross-sectional design of the study, we were unable to determine the causal direction of the relationships. Furthermore, data on service utilization or treatment for sleep disorders were not available, which may have been unmeasured confounding factors in the observed associations. The self-reported nature of insomnia symptoms that may be subject to recall and reporting bias is another important limitation of the study. We were also unable to conduct detailed analyses on the severity of the insomnia symptoms and differences in sleeping habits, which would have led to a better understanding of the association between insomnia symptoms and their covariates. The current study, on the other hand, allowed researchers to look at three types of sleep disorders and the correlates of experiencing any of these sleep disorders symptoms at older ages at the same time. The large sample of community-dwelling older people aged 60 years and above in a resource-constrained setting in India and the inclusion of several socio-demographic, lifestyle and medical variables are the major strengths of the study. Further, longitudinal and cause-effect studies are warranted to elaborate on the actual contribution of specific diseases and factors toward insomnia or vice versa.

## Conclusion

The findings of the study have important implications for understanding the nationwide prevalence and correlates of insomnia symptoms among older Indian adults. They show an association between being underweight, specific chronic diseases, and adverse lifestyle habits with insomnia symptoms among older adults. Thus, it is important to detect insomnia symptoms in older adults as early as possible and provide prompt treatment for all types of sleep problems. Policies that focus on promoting physical activity, better nutrition, and social involvement may enhance the quality and quantity of sleep and will improve the overall health of older individuals. Again, nutrition-based interventions and individual disease-specific management programs may help minimize the stressful situations in older people and develop a good night’s sleep for the older population.
